# Exploring the influence of artificial intelligence integration on personalized learning: a cross-sectional study of undergraduate medical students in the United Kingdom

**DOI:** 10.1186/s12909-025-07084-z

**Published:** 2025-04-17

**Authors:** Kehinde Sunmboye, Hannah Strafford, Samina Noorestani, Malena Wilison-Pirie

**Affiliations:** 1 University Hospitals of Leicester, Leicester, UK; 2 University of Leicester, Leicester, UK

**Keywords:** Artificial intelligence, Medical education, Personalized learning

## Abstract

**Background:**

With the integration of Artificial Intelligence (AI) into educational systems, its potential to revolutionize learning, particularly in content personalization and assessment support, is significant. Personalized learning, supported by AI tools, can adapt to individual learning styles and needs, thus transforming how medical students approach their studies. This study aims to explore the relationship between the use of AI for self-directed learning among undergraduate medical students in the UK and variables such as year of study, gender, and age.

**Methods:**

This cross-sectional study involved a sample of 230 undergraduate medical students from UK universities, collected through an online survey. The survey assessed AI usage in self-directed learning, including students’ attitudes towards AI accuracy, perceived benefits, and willingness to mitigate misinformation. Data were analyzed using descriptive statistics and linear logistic regression to examine associations between AI usage and demographics.

**Results:**

The analysis revealed that age significantly influenced students’ willingness to pay for AI tools (*p* = 0.012) and gender was linked to concerns about AI inaccuracies (*p* = 0.017). Female students were more likely to take steps to mitigate risks of misinformation (*p* = 0.045). The study also found variability in AI usage based on the year of study, with first-year students showing a higher reliance on AI tools.

**Conclusion:**

AI has the potential to greatly enhance personalized learning for medical students. However, issues surrounding accuracy, misinformation, and equitable access need to be addressed to optimize AI integration in medical education. Further research is recommended to explore the longitudinal effects of AI usage on learning outcomes.

**Supplementary Information:**

The online version contains supplementary material available at 10.1186/s12909-025-07084-z.

## Introduction

### The promise of artificial intelligence in education

Artificial Intelligence (AI) has become a transformative force across a wide range of sectors, and its integration into educational settings holds immense promise. In particular, AI has the potential to revolutionize personalized learning by delivering customized educational experiences tailored to the unique needs, preferences, and learning styles of individual students [1]. AI tools can analyze student performance data in real time, adjusting content delivery, difficulty levels, and even learning modalities based on students’ strengths and areas requiring further attention [2]. In the context of medical education, where the curriculum is both demanding and extensive, AI has the potential to enhance learning efficiency, optimize time management, and improve the depth of knowledge retention. Most discussions about the use of AI technologies can elicit varying degrees of fear and exhilaration in students [3].

In the field of Medical education, the use of AI tools is characterized by its ability to provide useful information in a prompt and timely manner especially when students or their teachers need to acquire theoretical knowledge [4].

AI-driven adaptive learning tools can help medical students manage this intense workload by offering personalized study plans, quizzes, and real-time feedback tailored to their specific needs [5]. By providing targeted reinforcement of knowledge and addressing gaps in understanding, AI can potentially enhance the effectiveness of learning while promoting greater engagement with complex medical content.

### Current landscape of AI in medical education

Despite the theoretical potential of AI, its actual integration into medical education remains limited and uneven across different educational settings. Recent studies have demonstrated both the opportunities and challenges associated with incorporating AI into medical curricula [6]. When used in Medical education, AI applications in education range from intelligent tutoring systems and predictive analytics to chatbots and virtual patient simulations [4]. These tools have been shown to improve learning outcomes by offering personalized feedback, enabling self-paced learning, and supporting student engagement with course materials. Moreover, AI has also been recognized for its ability to support educators by automating administrative tasks, such as grading and generating individualized learning reports [4].

However, the use of AI in medical education is not without its challenges. Concerns regarding the accuracy of AI-generated content, ethical considerations related to bias and data privacy, and the need for students to develop critical thinking skills in the face of increasingly automated systems are significant hurdles [7]. Furthermore, disparities in the access to AI technologies and the cost of some AI-driven learning tools may exacerbate existing inequalities within the student populations, with some students being better equipped to take advantage of these innovations than others [5].

While AI tools hold immense potential for transforming medical education, disparities in access to technology and prior exposure to AI may influence how effectively students adopt these tools. For instance, students from underprivileged backgrounds or those with limited prior experience using AI may face barriers to fully integrating these technologies into their learning routines [8]. Such disparities could exacerbate existing inequalities within the student population, making it essential to explore not only demographic factors like age and gender but also variables such as, year of study, technological accessibility and familiarity with AI systems factors can help educators design equitable strategies for AI integration that cater to diverse learner needs.

### Personalized learning and the technology acceptance model (TAM)

The Technology Acceptance Model (TAM) provides a useful framework for understanding how medical students adopt AI technologies for personalized learning [9]. According to TAM, two key factors drive technology adoption: perceived usefulness and perceived ease of use. In the context of AI integration, perceived usefulness refers to the extent to which students believe AI will improve their learning outcomes, such as enhancing their understanding of medical concepts or improving their exam performance. Perceived ease of use, on the other hand, reflects the degree to which students find AI tools intuitive and easy to integrate into their existing study habits.

Perceptions of AI’s usefulness and ease of use are not uniform across different demographic groups, indicating that more nuanced approaches may be needed to encourage widespread adoption of AI technologies in medical education [10].

### Rationale and importance of the study

The growing body of literature on AI in education, including in medical education, emphasizes the potential for AI tools to deliver transformative benefits. However, despite the theoretical promise of AI, relatively little is known about how medical students are currently using these technologies for self-directed learning, and even less is known about the factors that influence their attitudes toward AI adoption [11]. Understanding these patterns is critical for educators and developers aiming to optimize AI tools for educational use and ensure that students are equipped to use them effectively.

This study addresses this gap by exploring how different demographic factors, such as age, gender, and year of study, influence medical students’ use of AI for personalized learning.

The research questions guiding this study are:

1. How do age, gender, and year of study influence the adoption of AI tools for self-directed learning among undergraduate medical students?
2. What concerns do medical students have about the accuracy and reliability of AI-generated content, and how do these concerns vary across demographic groups?
3. How can AI tools be optimized to better meet the personalized learning needs of medical students at different stages of their education?

### Research significance and contribution to the field

This study makes several important contributions to the growing field of AI in education. First, by examining how different demographic groups perceive and use AI tools, this research provides valuable insights into the factors that drive or hinder AI adoption among medical students.

Second, this study sheds light on the ethical and practical concerns associated with AI use in education. In a medical education context, where errors can have serious implications, it is critical that students are taught how to critically evaluate AI-generated content and use these tools responsibly [12].

Finally, this study underscores the need for further research on how AI integration evolves over time as students’ progress through their medical education. While this study provides a snapshot of AI use at a single point in time, future longitudinal research is needed to understand how students’ attitudes toward AI change as they move from pre-clinical to clinical years. Such research could inform the development of AI tools that are better tailored to the specific needs of medical students at different stages of their academic journey.

## Methodology

### Study design

This study employed a cross-sectional quantitative research design, examining the relationship between AI usage and various demographic factors, such as age, gender and ethnicity. It also looked at year of study, at a single point in time. The cross-sectional approach allows for a snapshot of AI integration into the personalized learning strategies of medical students, providing insight into how different subgroups utilize AI and the associated factors influencing its adoption.

While cross-sectional studies are valuable for identifying associations, they do not establish causality. However, this design was deemed appropriate given the exploratory nature of this research, as it offers a cost-effective, time-efficient means of gathering preliminary data on AI usage patterns in medical education. A future longitudinal study may build on these findings to track changes in AI usage and attitudes over time.

### Setting and participants

The target population for this study was undergraduate medical students enrolled across various universities in the United Kingdom. Recruitment of participants was conducted via social media platforms, online medical student forums, and communities using convenience sampling, which facilitated broad accessibility and maximized participation. This approach enabled the inclusion of a diverse range of respondents from different institutions and backgrounds, ensuring representation across various levels of study and academic environments. Medical students were chosen because their unique learning environment demands the integration of vast amounts of theoretical and practical knowledge, making them ideal candidates for assessing the impact of AI on personalized learning.

While convenience sampling offers advantages such as ease of recruitment, cost-effectiveness, and rapid data collection, it also has limitations, including the potential for selection bias and reduced generalizability. To mitigate these concerns, efforts were made to reach a wide range of students from multiple universities and academic years. Additionally, demographic data were collected to assess the representativeness of the sample, and findings were interpreted with an awareness of these limitations. Jeong et al. [13] have shown that this approach is of value in mitigating the limitations of convenience sampling.

A total of 230 medical students voluntarily participated in the study. The target sample size was determined based on a power analysis conducted using G*Power software, indicating that a minimum of 200 responses would be necessary to achieve adequate statistical power [14]. This calculation assumed a medium effect size (f² = 0.15), an alpha level of 0.05, and a desired power of 0.80. Although the final sample size exceeded this threshold (*n* = 230), it remains relatively modest compared to the total population of UK medical students. Future studies should aim for larger samples to enhance generalizability and reduce sampling bias.

Efforts were therefore made to ensure diversity by recruiting participants through multiple social media platforms and university networks. However in spite of these efforts, the non-random nature of the sample limits the external validity of the findings, highlighting the need for caution when extrapolating these results to the wider, larger population of UK medical students.

### Survey development and instruments

The survey questionnaire was developed through a rigorous multi-step process involving consultation with experts in educational technology, AI, and medical education. Initially, a draft questionnaire was created based on a review of existing literature and frameworks related to AI adoption in education, including the Technology Acceptance Model (TAM) [9]. The initial version included both closed-ended and open-ended questions designed to capture quantitative data and qualitative insights.

A pilot study was conducted with a small group of 10 medical students to evaluate the clarity, structure, and relevance of the survey items. Feedback from the pilot group led to significant refinements, including rewording ambiguous questions and simplifying complex response options. For example, Likert-scale items were adjusted to ensure they aligned with the study’s objectives and were easily interpretable by participants. This iterative process ensured that the survey was straightforward, reduced ambiguity, and aligned with the study’s goals.

Validation of the questionnaire involved assessing its content validity and reliability. Content validity was established through expert review, with three independent reviewers evaluating each item for relevance and alignment with the study’s aims. Reliability testing was performed using Cronbach’s alpha, which yielded a satisfactory coefficient of 0.87, indicating strong internal consistency. These steps ensured the robustness and fit-for-purpose nature of the survey instrument.

The final survey comprised four sections:

#### Demographic information

Participants were asked to report their age, gender, year of study, and other background information such as prior experience with AI tools. This data enabled an analysis of how these factors influence AI usage patterns.

#### AI usage patterns

Participants were asked a series of questions about their engagement with AI tools for self-directed learning, including frequency of use, specific applications (e.g., content revision, quiz generation, or clinical simulations), and the types of AI tools they employed (e.g., chatbots, adaptive learning platforms).

#### Perceptions of AI

This section included questions on participants’ perceptions of AI’s effectiveness in enhancing learning, their concerns about AI accuracy, and their willingness to rely on AI for critical academic tasks. Questions also probed students’ attitudes toward AI misinformation and whether they took steps to mitigate it, such as cross-referencing AI-generated information with authoritative sources like clinical guidelines.

#### AI literacy and future integration

Questions focused on students’ awareness of AI’s limitations, ethical concerns, and their perceptions of how AI should be integrated into medical curricula in the future. This section also explored whether students felt they had been adequately trained to use AI tools effectively and ethically.

To assess AI usage, the survey incorporated a modified version of the AI Use Scale, a validated tool designed to measure the frequency and purposes of AI utilization across various educational activities. The scale included Likert-type items ranging from 1 (strongly disagree) to 5 (strongly agree) to capture attitudes and behaviours concerning AI usage.

This structured approach to survey development—initial item generation, expert consultation, piloting, refinement, and final validation—ensured that the final instrument was comprehensive, reliable, and aligned with the study’s objectives.

### Data analysis

Data analysis was performed using DATAtab^®^ statistical software. Both descriptive and inferential statistics were employed to examine the relationships between variables and to provide a comprehensive analysis of the findings.

#### Descriptive statistics

Descriptive statistics were used to summarize the demographic characteristics of the participants and their patterns of AI use. This included calculating means, standard deviations, and frequency distributions for variables such as age, gender, year of study, and AI use frequency.

#### Inferential statistics

To test for associations between independent variables (age, gender, year of study) and dependent variables (e.g., willingness to pay for AI, concern about AI misinformation, use of mitigation strategies), logistic regression and linear regression models were employed. These models were used to determine the strength and direction of relationships, with a *P*-value threshold of < 0.05 considered statistically significant.

#### Handling missing data

A rigorous approach was taken to handle missing data. Cases with incomplete responses were excluded from the inferential analysis but included in the descriptive analysis if sufficient information was provided for those sections. The impact of this exclusion on the final sample size and analysis results was minimal, as the majority of participants completed the survey in its entirety.

### Ethical considerations

This study adhered to ethical guidelines as set forth by the Research Ethics Committee of the University of Leicester. Ethical approval was obtained prior to data collection, and all protocols were followed to ensure the privacy and confidentiality of participant data. Informed consent was obtained electronically, with participants provided with detailed information regarding the study’s aims, procedures, and their rights as participants.

Confidentiality was maintained by anonymizing all data at the point of collection, with no identifying information stored or linked to survey responses. Data were securely stored in a password-protected database, accessible only to authorized research personnel. All data handling and analysis complied with GDPR standards and institutional regulations regarding the protection of personal information.

## Results

**Fig. 1:**
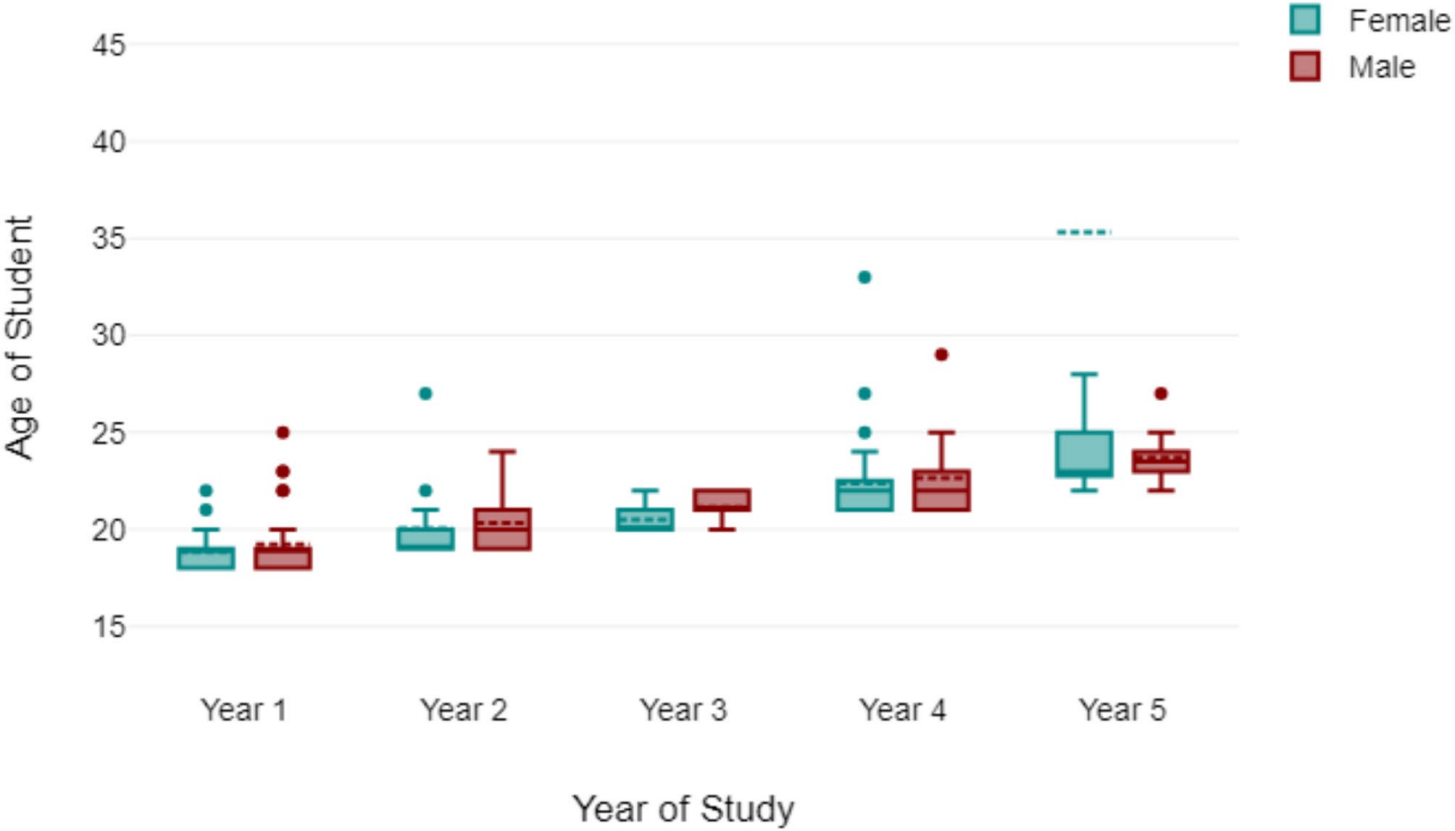
Box plot showing year of study and gender distribution

**Fig. 2:**
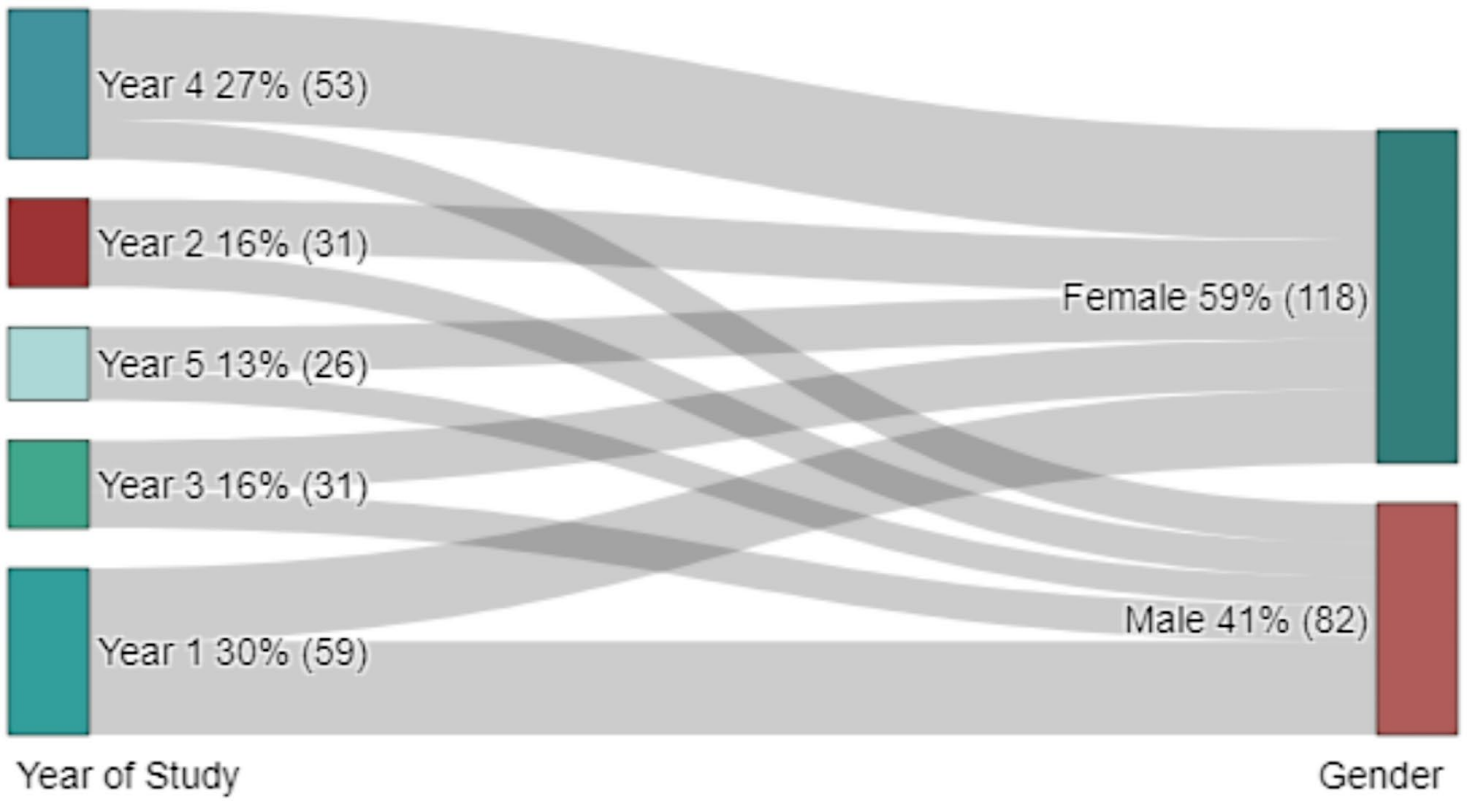
Sankey diagram showing proportion of students that rely on use of AI by gender and year of study

**Fig. 3:**
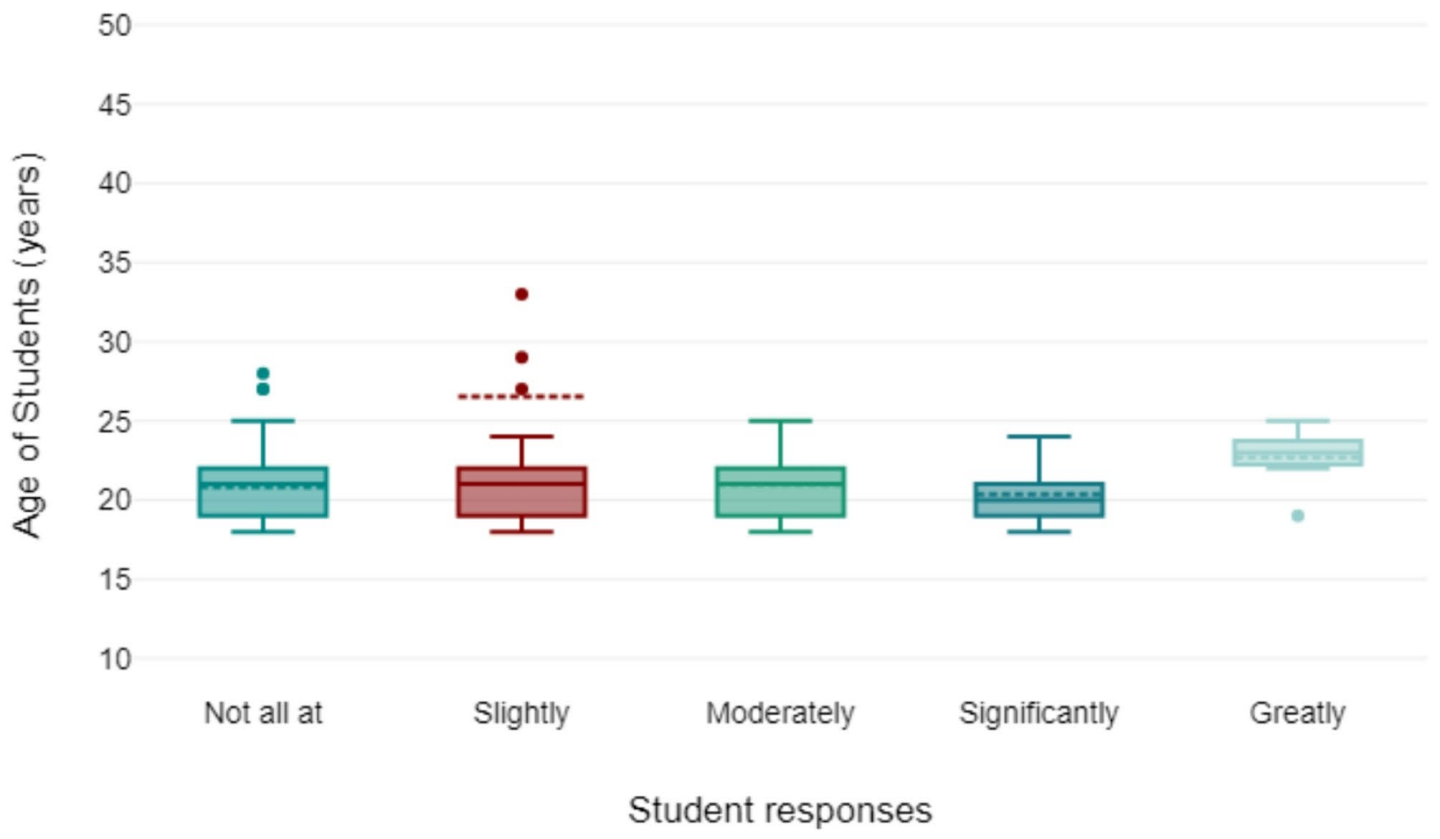
Box plot showing student responses by age to what extent has AI improved your self-directed learning

**Fig. 4:**
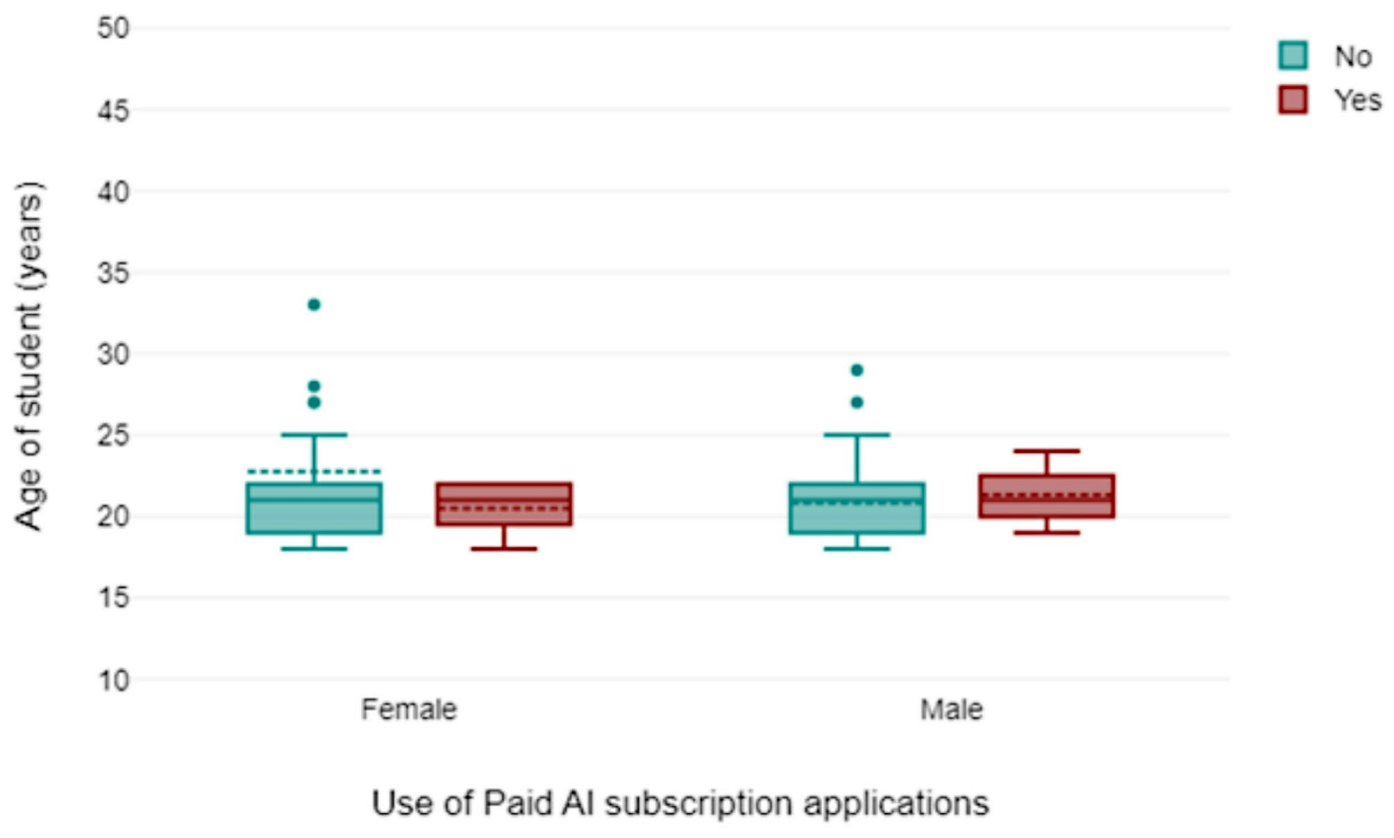
statement should read: Box plot showing gender distribution of paid subscriptions to AI applications made by age of students

**Fig. 5:**
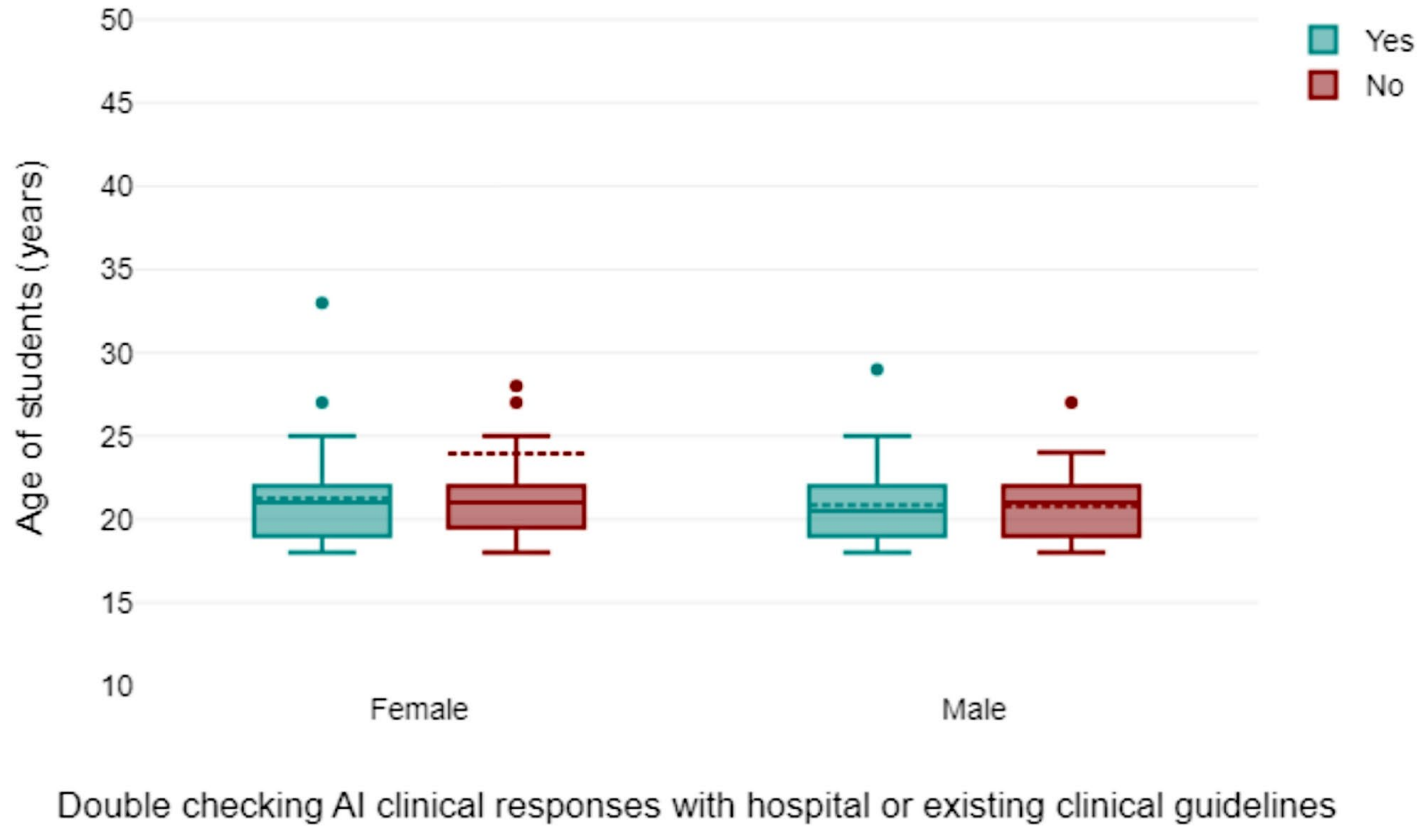
Box plot showing to gender distribution of students in relation to fact checking of AI responses with hospital or existing clinical guidance

230 responses were obtained from the online survey. Only 200 responses (87%) were used in the final analysis due to missing data or ambiguous responses. The age range of the students was from 19 years to 35 years (Fig. 1). Descriptive analysis showed that 59% of participants were female (Fig. 2), with AI usage varying significantly by year of study of the respondents (Fig. 2). First-year students reported the highest reliance on AI tools for self-directed learning, with usage decreasing among higher-year students. There was a spread of responses for students that reported that AI improved their learning (Fig. 3) and those students that paid for AI subscriptions (Fig. 4). There was also an even spread of responses between gender with students fact checking the AI responses obtained in their self-directed learning (Fig. 5).

The inferential statistical analysis (Table 1) identified significant associations between age and willingness to pay for AI subscriptions (*p* = 0.012), as well as gender and concerns about AI misinformation (*p* = 0.017). Female students were more likely to take steps to mitigate misinformation risks (*p* = 0.045).

**Table 1:** Table showing the *P*-values of the dependent variables following linear logistic regression

	*P*- values of the dependent variables					
Independent variables	Would you pay a subscription fee for continued access to AI for self-directed learning?	Do you consider the possibility of inaccuracies and misinformation provided by AI?	Do you try to mitigate against inaccuracies and misinformation provided by AI (for example by checking hospital guidelines)?	Do students using AI in self-directed learning gain an unfair exam advantage over those who don’t?	Are you concerned that other students using AI for self-directed learning may affect your relative exam performance?	Would it be beneficial for medical students to be taught how to maximise AI use for their self-directed learning?
Age of students	**0.012**	0.194	0.312	0.94	0.383	0.692
Year of study	0.486	0.427	0.469	0.171	0.092	0.849
Gender	0.05	**0.017**	**0.045**	0.766	0.288	0.494

## Discussion

This study examined the integration of Artificial Intelligence (AI) tools into the personalized learning strategies of undergraduate medical students in the UK, specifically analyzing how AI use correlates with variables such as age, gender, and year of study. The findings provide valuable insights into the varying levels of AI adoption across different demographic groups, and the potential factors driving these differences. The results also highlight key concerns regarding AI usage in educational contexts, particularly the accuracy and reliability of these tools, as well as students’ willingness to rely on AI for self-directed learning.

### Influence of age on AI usage

The study found a statistically significant relationship between age and students’ willingness to pay for AI subscription services (*p* = 0.012). Older students were more likely to invest in AI tools compared to younger counterparts. This may be attributed to the increased academic pressure faced by senior students as they approach clinical practice, with AI tools potentially offering an efficient solution to managing the volume of knowledge required in later years of medical education [15]. Older students might view AI as a tool for reinforcing knowledge and preparing for practical examinations, which are more prevalent in the later stages of medical training.

Younger students, particularly those in their first year, may not yet fully appreciate the utility of AI in facilitating deeper understanding, focusing instead on traditional study methods or introductory content. As first-year students reported the highest reliance on AI, this could indicate that newer students are experimenting with AI tools but may not yet be ready to invest financially in these technologies. The statistically significant finding that age influences willingness to pay for AI underscores the need for AI developers to consider cost-effective models student-focused pricing tiers, especially for younger students who might not be able to afford paid AI applications or do not believe it is a worthwhile investment towards their education.

### Gender differences in AI usage and perception of accuracy

Another notable result was the statistically significant relationship between gender and concerns about AI accuracy (*p* = 0.017). Female students exhibited a higher level of concern regarding potential inaccuracies in AI-generated content, and they were more likely to engage in behaviours aimed at mitigating these risks (*p* = 0.045). This gender disparity could be influenced by various factors, including differences in risk perception and information-processing styles between males and females [16].

Research suggests that women are more likely to engage in thorough cross-checking and validation of information in high-stakes contexts, such as in medical education, where accuracy is paramount [17]. This aligns with the study findings, as female students were more proactive in verifying AI outputs against external sources, such as clinical guidelines or hospital protocols. This behaviour demonstrates a more cautious approach to adopting AI technology in educational settings, possibly due to a heightened awareness of the potential consequences of relying on inaccurate information, especially in the medical field where errors can have serious implications.

Male students, while still using AI tools, did not express the same level of concern regarding accuracy and were less likely to take steps to mitigate potential misinformation from this study. This raises important questions about how AI systems present information to users and whether certain user groups require more targeted education on how to effectively utilize AI while minimizing risks.

### Impact of year of study on AI usage

While the study did not find a statistically significant relationship between the year of study and many of the dependent variables (*p*-values for most were above 0.05), descriptive data indicated that first-year students relied more heavily on AI tools for their learning compared to those in later years. This reliance may reflect the novelty of AI for newer students, who are still adjusting to the demands of medical education and are eager to leverage new technologies to enhance their study routines. First-year students, still in the process of building foundational knowledge, may find AI tools helpful for organizing, synthesizing, and revisiting large volumes of introductory content.

In contrast, students in their later years, particularly those approaching clinical placements, may have developed more established study habits that rely less on AI and more on practical, hands-on experiences. As they progress through their education, these students may prioritize clinical skills and face-to-face instruction over AI-powered learning, viewing AI as more supplementary than essential.

The absence of a statistically significant link between year of study and AI use raises interesting possibilities for future research. It suggests that while AI tools may be widely available, their adoption is not necessarily uniform across academic levels. This finding highlights the need for tailored AI solutions that evolve alongside students’ educational journeys, offering different functionalities to meet the changing demands of medical education as students’ progress from theoretical learning to clinical application.

### Ethical concerns and educational implications

The study revealed that AI’s role in personalized learning is not without its challenges, particularly regarding ethical considerations. Both the literature and this study’s findings indicate that AI-driven tools carry the risk of propagating misinformation, and there is a significant level of concern among students, particularly female students, about the accuracy of AI-generated content. These concerns are not unfounded, as AI systems, particularly those driven by machine learning, can reflect the biases present in their training data (Thomas., 2019). If not properly mitigated, such biases could lead to the reinforcement of incorrect or harmful medical knowledge.

This issue is particularly concerning in the context of medical education, where accuracy is crucial. Misleading AI outputs could have serious consequences, potentially affecting students’ understanding of key medical concepts and, ultimately, their ability to provide accurate patient care in the future. Therefore, it is critical for AI developers and educational institutions to implement safeguards that ensure the reliability of AI systems used in medical training.

One potential solution is to incorporate AI literacy into medical education curricula, ensuring that students are equipped with the skills needed to critically evaluate AI-generated content. This training could help students understand the limitations of AI and develop strategies for cross-referencing AI outputs with trusted medical resources [18]. Additionally, medical institutions could collaborate with AI developers to improve the accuracy and transparency of AI systems, ensuring that these tools provide reliable support for students’ learning.

### Access to technology and AI tools

Beyond variables such as age, gender, and year of study, other factors likely play a role in shaping students’ attitudes toward AI. For instance, prior exposure to AI technologies and access to reliable internet connectivity may significantly influence how readily students embrace AI tools for personalized learning [19]. Students who have grown up in technologically advanced environments or received formal training in AI literacy may feel more confident in leveraging these tools effectively [20]. Conversely, those without such advantages may struggle to integrate AI into their learning routines, potentially widening the gap between high- and low-performing students [19]. Addressing these disparities will require targeted interventions, such as providing subsidized access to AI tools and offering introductory workshops on AI basics.

### Limitations and future research

Several limitations should be considered when interpreting the results of this study. First, while convenience sampling introduces potential concerns regarding generalizability, it remains a widely used method for obtaining initial insights into emerging fields such as AI in medical education. The study recruited participants from multiple online medical student forums, university networks, and social media platforms, ensuring a broad range of respondents from various institutions. However, as non-random sampling can increase the risk of selection bias, future research should employ stratified random sampling to enhance the representativeness of findings across the broader population of UK medical students.

Secondly, the reliance on self-reported data introduces the potential for social desirability and recall bias, as participants may overstate their engagement with AI tools or underreport concerns about AI accuracy. To mitigate these biases, future research should complement self-reported data with objective measures such as learning analytics, AI interaction logs, or experimental studies that track real-time AI usage patterns [21]. Additionally, qualitative interviews combined with observational data could provide deeper insights into students’ engagement with AI in self-directed learning [22, 23].

The cross-sectional design of this study also limits its ability to draw conclusions about changes in AI use over time. While this design provides a useful snapshot of AI integration at a single point, it does not capture longitudinal trends or shifts in students’ attitudes and behaviours as they progress through medical training. Future studies should adopt a longitudinal approach to track AI adoption from pre-clinical through clinical years, identifying specific stages where AI literacy interventions may be most beneficial.

Beyond demographic factors, AI adoption may also be influenced by students’ access to technology, digital literacy levels, and prior exposure to AI tools. Studies have shown that disparities in digital access and proficiency can impact students’ ability to effectively use AI-driven learning resources [24]. Future research should explore how these factors contribute to AI adoption and whether institutional interventions, such as AI literacy workshops, can help bridge existing gaps [22, 21].

Future research should also examine the impact of AI on specific educational outcomes, such as academic performance, knowledge retention, and the development of clinical reasoning skills. By focusing on these outcomes, researchers can better understand the true value of AI in medical education and identify strategies for optimizing its integration into personalized learning frameworks [21]. Additionally, investigating how AI literacy training can be effectively incorporated into medical curricula will be essential in preparing students for the evolving role of AI in clinical practice [23].

## Conclusion

This study provides critical insights into the role of AI in personalized learning among undergraduate medical students in the United Kingdom. The findings of this study underscore the potential of AI to enhance learning in medical education, but also emphasize the need for institutions to address ethical challenges such as equity in access and the accuracy of AI-generated information. Medical schools should integrate AI literacy into their curricula, ensuring students are equipped to critically evaluate AI outputs while promoting fair and responsible use.

By addressing these gaps, the study contributes to a deeper understanding of how AI can be effectively and ethically integrated into medical education, ultimately supporting the development of future healthcare professionals who can leverage AI tools in a clinical context.

## Electronic supplementary material

Below is the link to the electronic supplementary material.

Supplementary Material 1

## Data Availability

The datasets generated and/or analyzed during the current study are available from the corresponding author upon reasonable request.
