# Supplementary material for: Exploring the influence of artificial intelligence integration on personalized learning: a cross-sectional study of undergraduate medical students in the United Kingdom
Source: BMC Med Educ. 2025 Apr 17;25:570. doi: 10.1186/s12909-025-07084-z (PMC12007362; doi:10.1186/s12909-025-07084-z)
Supplement: Supplementary file 1 — Supplementary Material 1 [file 12909_2025_7084_MOESM1_ESM.pdf]

Questionnaire: The Use of AI in Personalized Learning Among Medical Students

Section 1: Demographic Information

1. What is your age? (Open-ended)
2. What is your gender?
   - Male
   - Female
   - Prefer not to say
3. What year of study are you currently in?
   - First year
   - Second year
   - Third year
   - Fourth year
   - Fifth year
4. Have you had any prior experience using AI tools in your education?
   - Yes
   - No
5. If yes, which AI tools have you used? (Select all that apply)
   - Chatbots (e.g., ChatGPT)
   - Adaptive learning platforms (e.g., Quizlet, Smart Sparrow)
   - Virtual patient simulations
   - AI-powered revision tools
   - Other (Please specify)

Section 2: AI Usage Patterns

6. How often do you use AI tools for self-directed learning?
   - Daily
   - Weekly
   - Monthly
   - Rarely
   - Never
7. For which educational tasks do you primarily use AI tools? (Select all that apply)
   - Content revision
   - Quiz generation
   - Clinical simulations
   - Research and literature reviews
   - Time management and study planning
   - Other (Please specify)
8. What motivates you to use AI in your learning? (Select all that apply)
   - To improve efficiency
   - To personalize learning to my needs
   - To access real-time feedback
   - To reduce study time
   - Other (Please specify)
9. Are you currently paying for any AI tools to support your education?
   - Yes
   - No

10. Would you be willing to pay for AI tools in the future to support your self-directed learning?
    - Yes
    - No

Section 3: Perceptions of AI in Learning

11. To what extent do you agree with the following statements about AI tools in your learning? (Likert scale: 1 - Strongly disagree to 5 - Strongly agree)
    - AI tools improve my understanding of medical concepts.
    - AI helps me organize my study schedule.
    - AI enhances my exam preparation.
    - I trust the accuracy of information provided by AI tools.
    - I rely on AI tools for important academic tasks.
12. How concerned are you about the possibility of AI-generated content being inaccurate or misleading?
    - Not concerned
    - Slightly concerned
    - Moderately concerned
    - Very concerned
13. Have you ever encountered inaccurate information from AI tools during your studies?
    - Yes
    - No
14. How often do you fact-check AI-generated information against trusted sources (e.g., textbooks, clinical guidelines)?
    - Always
    - Sometimes
    - Rarely
    - Never
15. Are there specific types of content that you avoid using AI for due to concerns about accuracy? (Open-ended)

Section 4: AI Literacy and Ethical Considerations

16. How familiar are you with the ethical implications of using AI in medical education? (Likert scale: 1 - Not familiar at all to 5 - Very familiar)
17. Do you believe AI tools in education may reinforce biases or ethical issues?
    - Yes
    - No
18. Do you think medical students should be taught how to critically evaluate AI-generated content?
    - Yes
    - No
19. Do you feel adequately trained to use AI tools effectively and ethically in your learning?
    - Yes
    - No
20. In your opinion, how should AI be integrated into medical curricula in the future? (Open-ended)

Section 5: Future Considerations and Feedback

21. Do you believe AI tools provide an unfair advantage to students who can afford paid subscriptions?
    - Yes
    - No
22. Would you like to see more AI tools integrated into your medical education?
    - Yes
    - No
23. What improvements or changes would you suggest for AI tools in medical education? (Open-ended)
